# Biomechanics of TGFβ-induced epithelial-mesenchymal transition: implications for fibrosis and cancer

**DOI:** 10.1186/2001-1326-3-23

**Published:** 2014-07-15

**Authors:** Joseph W O’Connor, Esther W Gomez

**Affiliations:** 1 Department of Chemical Engineering, The Pennsylvania State University, 204 Fenske Laboratory, 16802 University Park, PA, USA; 2 Department of Biomedical Engineering, The Pennsylvania State University, 16802 University Park, PA, USA

**Keywords:** Epithelial-mesenchymal transition, Biomechanics, Mechanotransduction, Myofibroblast, Fibrosis, Cancer, Transforming growth factor, Cell shape, Matrix rigidity

## Abstract

Fibrosis, a disease that results in loss of organ function, contributes to a significant number of deaths worldwide and sustained fibrotic activation has been suggested to increase the risk of developing cancer in a variety of tissues. Fibrogenesis and tumor progression are regulated in part through the activation and activity of myofibroblasts. Increasing evidence links myofibroblasts found within fibrotic lesions and the tumor microenvironment to a process termed epithelial-mesenchymal transition (EMT), a phenotypic change in which epithelial cells acquire mesenchymal characteristics. EMT can be stimulated by soluble signals, including transforming growth factor (TGF)-β, and recent studies have identified a role for mechanical cues in directing EMT. In this review, we describe the role that EMT plays in fibrogenesis and in the progression of cancer, with particular emphasis placed on biophysical signaling mechanisms that control the EMT program. We further describe specific TGFβ-induced intracellular signaling cascades that are affected by cell- and tissue-level mechanics. Finally, we highlight the implications of mechanical induction of EMT on the development of treatments and targeted intervention strategies for fibrosis and cancer.

## Introduction

Fibrotic diseases promote loss of function in a variety of organs including the heart, liver, lung, and kidney resulting in a significant number of deaths worldwide [1,2]. Fibrosis arises from deregulation of wound healing processes and is characterized by a stiff and collagen-rich extracellular matrix (ECM) that is resistant to degradation. Inappropriate activation and activity of myofibroblasts drives the development of fibrosis. An increased risk of developing cancer in a variety of tissues has been linked to high stromal collagen content and to the presence of fibrotic lesions [3-5]. Indeed, fibrosis has been found in close proximity of tumors within the pancreas [6], liver [7], and kidney [8] and myofibroblasts have been identified as residents of the tumor microenvironment [9,10]. The purpose of this article is to review the role of myofibroblasts in fibrosis and cancer and to discuss how physical cues contribute to epithelial-mesenchymal transition and to the development of myofibroblasts.

## Review

### Myofibroblasts in health and disease

Myofibroblasts are specialized cells within the body that aid in wound healing. Upon activation by biochemical and mechanical signals, myofibroblasts secrete and organize ECM, develop specialized matrix adhesions [11], and exhibit cytoskeletal organization characterized by contractile actin filaments [12]. Together, these features allow for re-establishment of mechanical integrity and stability to the damaged tissue and enable the myofibroblasts to exert large contractile forces on their microenvironment thus assisting in both the closure of the wound and remodeling of the tissue [13,14]. When wound healing is complete, myofibroblasts undergo apoptosis which decreases the cellularity of the granulation tissue and promotes the formation of scar tissue [15]. Due to their important role in wound healing, these cells have attracted much interest for regenerative medicine applications.

Upon aberrant and chronic activation, myofibroblasts can mediate the development of fibrosis [16-20]. The sustained activation of myofibroblasts results in the enhanced production of ECM components, including collagen types I, III, IV, V, and VI and fibronectin [21-23]. Through integrin engagement with ECM components and cytoskeletal contractility, myofibroblasts exert large forces on the ECM thus enabling matrix assembly and alignment [24]. Together, these factors can lead to stiffening of the tissue and disruption of tissue architecture and function.

Studies suggest that myofibroblasts are key players in the progression of a variety of cancer types including lung [5], liver [3,25], breast [26], and gastric [27] cancer. Myofibroblasts have been found at the invasive fronts of tumors where they secrete pro-invasive cytokines, proteases, and inflammatory mediators [28-36]. Fibrotic lesions and myofibroblasts have also been found in the tumor microenvironment prior to cancer cell invasion into the stroma suggesting that myofibroblasts may mediate an invasive phenotype [37]. Indeed, myofibroblasts are found in higher proportions in the stroma of invasive breast cancers than in *in situ* carcinoma and their presence has been correlated with lymph node metastasis and increased histological grade in invasive ductal carcinoma [38,39]. Contrary to these reports, a recent study found that myofibroblasts may serve a protective role in the context of pancreatic cancer as depletion of myofibroblasts and fibrosis in a mouse model of pancreatic ductal adenocarcinoma leads to a more invasive cancer cell phenotype and reduced survival [40]. Depletion of myofibroblasts promoted remodeling of the pancreatic tumor stroma as well as changes in immune cell infiltration to the tumor. Thus, the effect of myofibroblasts on cancer progression appears to be complex and multifaceted and may vary depending upon organ or stage of cancer. Future investigation of the effect of *in vivo* myofibroblast depletion in other cancer types will be informative and will shed further light on the role of myofibroblasts in tumor progression.

### Differentiation of myofibroblasts from fibroblasts

Following the identification of myofibroblasts, studies focused primarily on factors that regulate the differentiation of myofibroblasts from stromal fibroblasts. Transforming growth factor (TGF)-β is a potent inducer of the myofibroblast phenotype and TGFβ-induced differentiation of fibroblasts to myofibroblasts was found to depend on the ED-A domain of fibronectin [41]. Hallmarks of differentiated myofibroblasts include *de novo* expression of alpha smooth muscle actin (αSMA) and the incorporation of αSMA into stress fibers which confers high contractile activity to myofibroblasts. In this process, mechanical tension is crucial for the development of contractile features and for the acquisition of a myofibroblast phenotype [42-46]. Culture of fibroblasts on two-dimensional compliant substrata or within three-dimensional collagen gels has revealed that increased microenvironmental stiffness and tension results in increased differentiation of fibroblasts to myofibroblasts [43,47]. *In vitro* and *in vivo* experiments have also shown that changes in tensional loads to either collagen gels with embedded fibroblasts or granulation tissue at wound sites results in altered contractility of myofibroblasts [48,49]. Likewise, myofibroblasts can actively remodel both the chemical and physical properties of their microenvironment through the secretion of ECM and exertion of contractile forces [50]. Thus, mechanical signals are essential for the development of myofibroblasts from fibroblasts and for proper physiological function.

### Epithelial cells mediate fibrogenesis

**Figure 1:**
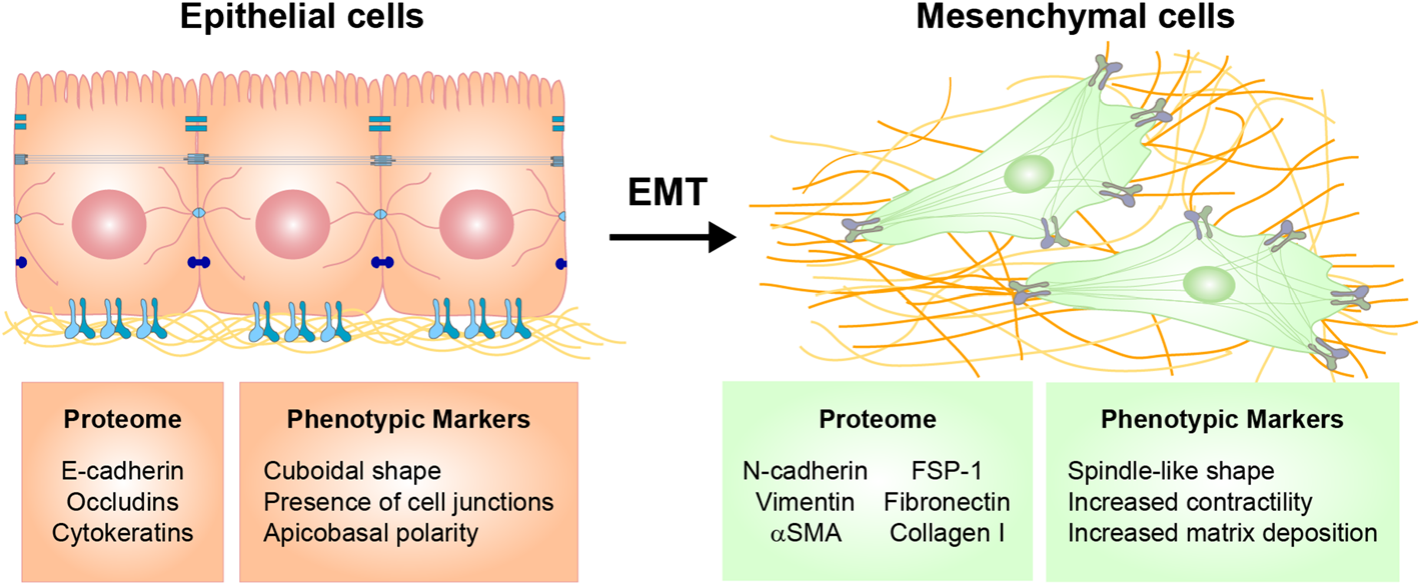
**Schematic representation of epithelial-mesenchymal transition.** EMT is a process in which epithelial cells disaggregate and exhibit dramatic shape changes. The transitioning epithelial cells lose polarity and intercellular contacts and gain mesenchymal properties such as increased migratory capacity, contractility, and production of extracellular matrix proteins. Common protein markers of epithelial and mesenchymal phenotypes are listed.

Epithelial-mesenchymal transition (EMT) is a form of epithelial plasticity that is important in normal embryonic development and is co-opted in the progression of pathological conditions including fibrosis and cancer [51-54]. In EMT, epithelial cells, which form monolayers that line many body structures and compartments, loosen attachments to neighboring cells, acquire an elongated morphology, and display increased motility (Figure 1). In addition to these phenotypic shifts, cells exhibit alterations in gene expression including upregulation of a variety of transcription factors including Snail, Slug, and Twist, decreased expression of epithelial markers such as E-cadherin and cytokeratins, and *de novo* expression of mesenchymal markers such as N-cadherin and vimentin [51,55]. Following early EMT marker changes, further progression through EMT can stimulate a myogenic program characterized by the expression of αSMA and a myofibroblast phenotype [56]. EMT is believed to contribute to fibrogenesis by serving as a source of myofibroblasts and by promoting paracrine signaling between epithelial cells and stromal cells. Several recent reviews highlight the role of EMT in epithelial-mesenchymal interactions in the context of fibrotic diseases [57-59].

Many studies have identified cells demonstrating features of EMT in fibrotic disease models and in human biopsy samples. In a model of liver fibrosis, hepatocytes upregulate the expression of the EMT-associated transcription factor Snail and hepatocyte-specific ablation of Snail protects mice from fibrotic progression [60]. In this study, it was found that hepatocyte expression of Snail has a multifaceted effect on the progression of liver fibrosis through regulation of growth factor expression and ECM synthesis, which impacts hepatocytes themselves and other cell types. Furthermore, hyperplastic type II alveolar epithelial cells from patients suffering from idiopathic pulmonary fibrosis co-express epithelial and mesenchymal markers including αSMA [61-63]. In addition, human renal biopsy samples from patients with a variety of fibrotic kidney diseases display cells within the tubular structures that exhibit both epithelial and mesenchymal features [64,65]. Together, these findings lend strong support for an important role for the epithelium in fibrogenesis either as a precursor of myofibroblasts or as a mediator influencing the development of myofibroblasts from other cell types.

Given the importance of myofibroblasts in health and disease, much effort has been directed toward identification of myofibroblast progenitor cell types through lineage tracing studies in *in vivo* disease models. Studies have revealed a variety of candidates including resident fibroblasts, mesenchymal stem cells, and endothelial cells, with a number of reports finding that a portion of myofibroblasts within fibrotic lesions arise from epithelium through EMT [27,61,66-71]. Fate tracking of alveolar epithelial cells in genetically modified mice has demonstrated that mesenchymal cells arising during the progression of pulmonary fibrosis can originate from epithelial cells [61,69,70]. Moreover, lineage tracing in animal models has also identified epithelial cells as one of the cell types from which myofibroblasts can originate in kidney, liver, and intestinal fibrosis with the proportion of myofibroblasts arising from epithelial cells being tissue specific [66,71,72]. However, a series of recent fate mapping studies using different epithelial and mesenchymal tags showed no evidence of epithelial precursors to myofibroblasts in the kidney and liver suggesting an alternate precursor cell type or that the role of the epithelium in fibrogenesis may be organ or disease specific [64,73-77]. Thus, whether epithelial cells are indeed a source of myofibroblasts *in vivo* is currently debated and yet to be resolved definitively. Several recent reviews provide a summary of the different viewpoints with regard to this debate [58,64,73].

### Biochemical induction of EMT by TGFβ

EMT is triggered by a variety of soluble factors including epidermal growth factor (EGF), hepatocyte growth factor (HGF), fibroblast growth factor (FGF), and TGFβ [55,78-85]. Other stimuli, such as hypoxia [86,87] and adhesion to ECM components can also induce EMT [69,88,89]. TGFβ, a ubiquitously expressed cytokine, is a potent inducer of EMT and is regarded as a key mediator of wound healing [90,91], fibrosis [92,93], and cancer [94,95]. Epithelial cells derived from a variety of tissues including lung [69,96,97], kidney [98-101], and breast [85,102-104] display myofibroblast features following exposure to TGFβ. TGFβ is perhaps the best characterized promoter of EMT and therefore we will focus this review specifically on TGFβ-mediated EMT.

In the canonical TGFβ signaling pathway, active TGFβ ligands initiate signaling by binding to type I and type II receptor serine/threonine kinases. Following receptor activation, Smad2 and Smad3 associate with the TGFβ receptor complex and are phosphorylated by the type I TGFβ receptor. Phosphorylated Smad2 and Smad3 then form a complex with Smad4 and translocate to the nucleus. Once in the nucleus, the Smads can regulate the transcription of target genes in conjunction with other nuclear co-factors [105-107]. Activation of several Smad-independent pathways including phosphoinositide 3-kinase (PI3K)-Akt [108], focal adhesion kinase (FAK) [109], p38 mitogen-activated protein kinase (p38MAPK) [110], and extracellular signal-regulated kinase (Erk) [111] have been identified as crucial for EMT induction by TGFβ and recent studies implicate hyaluronan synthase 2 (HAS2) [112], Krüppel-like factor (KLF)-8 [113], and microRNA miR-203 [114] as critical regulators of EMT. During the progression of TGFβ-induced EMT, cells exhibit dramatic cytoskeletal reorganization that is mediated by signaling through the Rho GTPase pathway which stimulates stress fiber formation, the acquisition of a mesenchymal morphology, and increased cytoskeletal contractility. Evidence implicates the RhoA pathway as a necessity for induction of EMT by TGFβ [108,115]. These changes in cell morphology and cytoskeletal architecture suggest an important role for physical cues in regulating EMT.

### Mechanical activation of TGFβ

TGFβ is synthesized by cells and stored in a latent form crosslinked to the ECM in the cellular microenvironment. TGFβ can be activated via a number of mechanisms, one of which is through integrin binding. Integrin α_v_β_6_, which is expressed at high levels predominantly on injured epithelial cells or cancer cells [116], binds to and locally activates TGFβ *in vivo* and *in vitro*[117]. Treatment of lung epithelial cells with cytochalasin D, an inhibitor of actin polymerization, blocks activation of TGFβ by α_v_β_6_ demonstrating an important role for the actin cytoskeleton in inducing TGFβ bioactivity [117]. A recent study suggests that cellular contractility is required for TGFβ activation by α_v_β_6_ as treatment of lung epithelial cells with Y27632 or blebbistatin, which inhibit Rho associated kinase (ROCK) and non-muscle myosin II respectively, abrogates TGFβ activation [118]. Myofibroblasts can also activate TGFβ through a combination of α_v_β_5_ and α_v_β_3_ integrin engagement and contractile forces *in vitro*[119]. Thus, cytoskeletal tension and cellular force generation are key mediators of the activation of TGFβ signaling.

### EMT alters the mechanical properties of cells

Cellular mechanics are influenced in part by the combination of cell morphology and cytoskeletal organization with the formation of stress fibers enabling increased cellular contractility [120]. Atomic force microscopy (AFM) is a useful tool to determine mechanical properties through gently applying a force to induce cell deformation from which the modulus of the cell can be determined. By employing AFM on kidney [121], alveolar [122], and mammary [123] epithelial cells, researchers have identified a significant increase in the stiffness of cells following TGFβ treatment. Tension within the membrane, as determined by tether pulling experiments, was also found to increase after EMT induction [123]. In addition, the topography of cells changes following treatment with TGFβ with a rougher surface profile [122] and nodular protrusions at intercellular junctions accompanying the transition to a mesenchymal phenotype [121]. These mechanical changes, in addition to cytoskeletal rearrangements, demonstrate a correlation between cytoskeletal architecture and increased cell stiffness as epithelial cells progress through EMT. Furthermore, EMT has been observed at the edges of epithelial wounds [101,124] and AFM studies have found that cell stiffness peaks approximately 10-20 μm from the wound edge with lower localized mechanical stiffness at the wound edge and far from the wound edge within the intact epithelial monolayer [125]. This peak in mechanical stiffness was nullified with the expression of a dominant negative form of RhoA. These data suggest that wound sites may serve as focal points for mechanical signaling events and that changes in cellular stiffness may provide signals for cellular processes including cell spreading and migration which are required for the early stages of epithelial wound healing.

### Increased cell spreading and elongation promote EMT

The shape of a cell is regulated by microenvironmental cues and has been shown to play a pivotal role in tissue morphogenesis [126,127], proliferation [128,129], apoptosis [128], and differentiation [130-133]. Cell shape is a consequence of intrinsic cellular mechanical properties and of forces exerted on the cell due to its adhesion to environmental components including ECM proteins and neighboring cells [134,135]. During EMT, cells experience drastic shape changes as they transition from a cuboidal, cobblestone morphology characteristic of epithelial cells to an elongated, spindle-like shape typical of mesenchymal cells.

**Figure 2:**
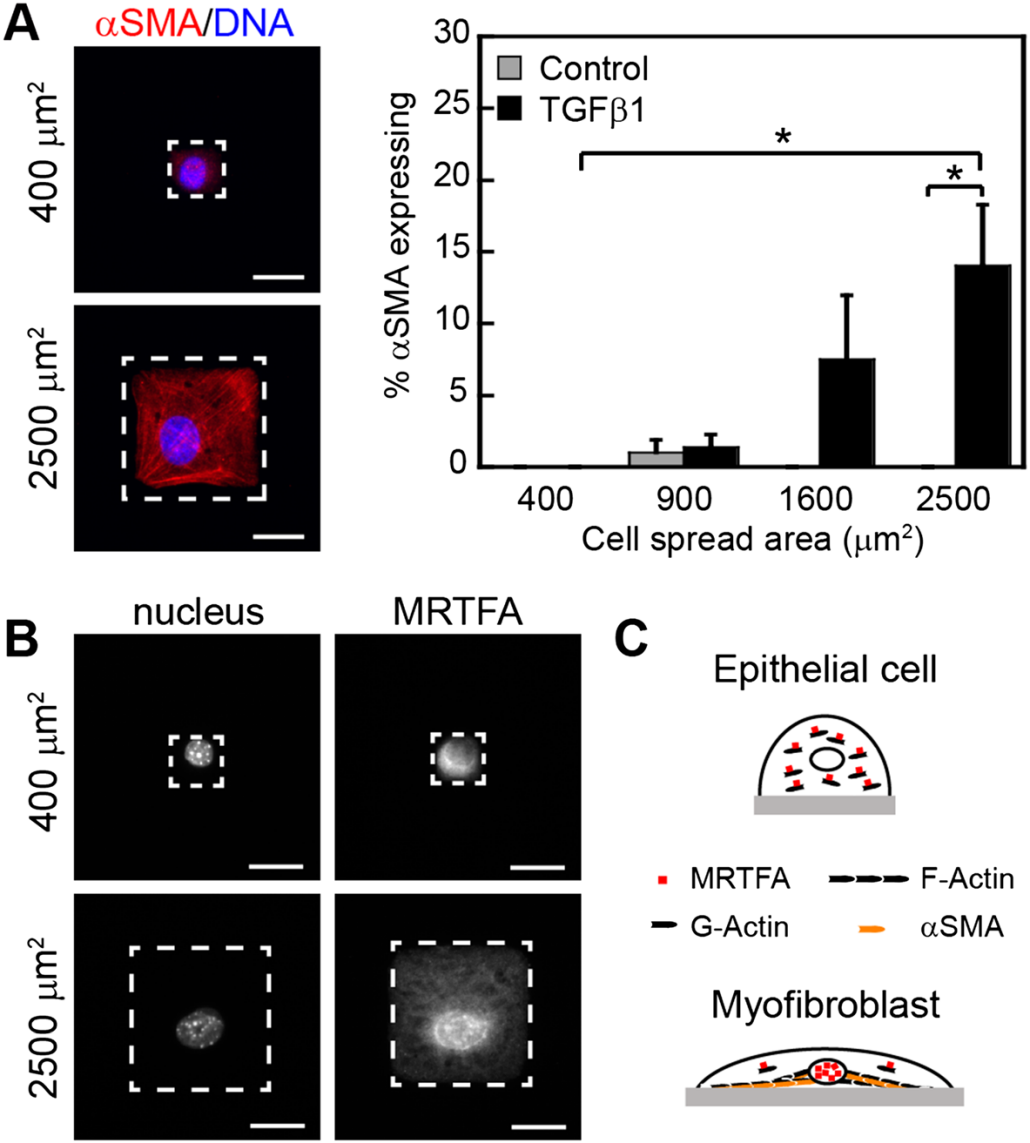
**Cell shape regulates epithelial-myofibroblast transition. (A)** Immunofluorescence staining and quantification of TGFβ-induced αSMA expression for mouse mammary epithelial cells cultured on 400 μm^2^ and 2500 μm^2^ fibronectin islands. The percentage of cells expressing αSMA following a 48 hour treatment with TGFβ or control vehicle was determined by immunofluorescence staining and microscopy. Cells with fluorescence intensities above background levels were scored as expressing αSMA. **(B)** Immunofluorescence staining for MRTFA in TGFβ-treated NMuMG cells shows increased nuclear localization of MRTFA when cells are permitted to spread (2500 μm^2^) in comparison to when cell spreading is blocked (400 μm^2^). MRTFA localization was determined by comparing the mean nuclear and cytoplasmic fluorescence intensities within cells. Dashed lines represent the perimeter of the cell. Scale bars, 20 μm. Reported values are the mean of three independent experiments ± standard error of the mean. *p < 0.05. **(C)** Proposed model demonstrating how cell spreading affects MRTFA subcellular localization and myofibroblast development. Adapted from O’Connor and Gomez, 2013 [104].

Through the use of micropatterned cell culture substrata, which enable precise control over cell spreading, studies have shown that cell shape regulates the expression levels of the epithelial marker cytokeratin and the mesenchymal marker vimentin in matrix metalloproteinase (MMP)-3-induced EMT but not in TGFβ-induced EMT [103]. More recently, we have demonstrated that cell spreading and elongation are critical factors that regulate TGFβ-induced expression of the myofibroblast marker αSMA during EMT [104]. Culturing epithelial cells on microcontact printed islands of fibronectin of varying sizes and shapes enabled control of cell morphology. Adhesion to large square islands (2500 μm^2^) which permitted cells to spread promoted an increase in the percentage of cells expressing αSMA after 48 hours of TGFβ treatment in comparison to cells blocked from spreading (400 μm^2^) and to control cells not treated with TGFβ (Figure 2A). We found that cell shape regulates αSMA expression in part by controlling the subcellular localization of myocardin related transcription factor (MRTF)-A (Figure 2B). MRTFA is a co-factor of serum response factor (SRF) and together these proteins regulate the transcription of a variety of genes associated with actin dynamics and cell contractile function including αSMA [136,137]. Indeed, MRTFA plays a key role in TGFβ-induced EMT [138] and contributes to experimental fibrosis [139] and metastasis [140]. The activity of MRTFA is regulated in part by its association with monomeric (G)-actin and polymerization of actin monomers into filamentous (F)-actin disrupts the association between MRTFA and G-actin thus enabling nuclear accumulation of MRTFA [141]. Increased cell spreading promotes an increase in F-actin levels which then leads to MRTFA nuclear localization and transcriptional activity (Figure 2C). These data suggest that cell shape changes that accompany EMT are critical for induction of the myofibroblast phenotype.

### Matrix rigidity controls EMT

Microenvironmental physical properties, such as stiffness and tension, are becoming increasingly acknowledged as contributing to normal cellular processes and to the development of diseases [142-145]. In wound healing, fibrosis, and cancer, epithelial cells exist in a heterogeneous microenvironment in which the chemical and mechanical properties are dynamic. For example, during wound healing the mechanical properties at the wound site evolve with time, from compliant (with a Young's modulus of approximately 1 kPa) after initial wounding to a stiffness of 25 kPa or greater for contracting wound granulation tissue [146]. In fibrotic tissues, the elastic modulus can reach values as high as 15-100 kPa [147-150]. Interestingly, a recent study found that increased microenvironmental rigidity may precede liver fibrosis suggesting that the mechanical properties of the matrix may promote activation of pro-fibrotic pathways [143,151]. *In vivo* and *in vitro* studies have also linked increased tissue stiffness and collagen content to the tumor phenotype and metastasis [144,145,152]. For example, during the progression of breast cancer, the stiffness of the mammary gland can range from approximately 200 Pa for normal tissue to 5000 Pa or greater for the average breast tumor [145,149,153]. High mammographic density, a strong risk factor for breast cancer [154,155], is associated with a significantly greater collagen content within the mammary gland in comparison to breast tissue with less mammographic density [4].

**Figure 3:**
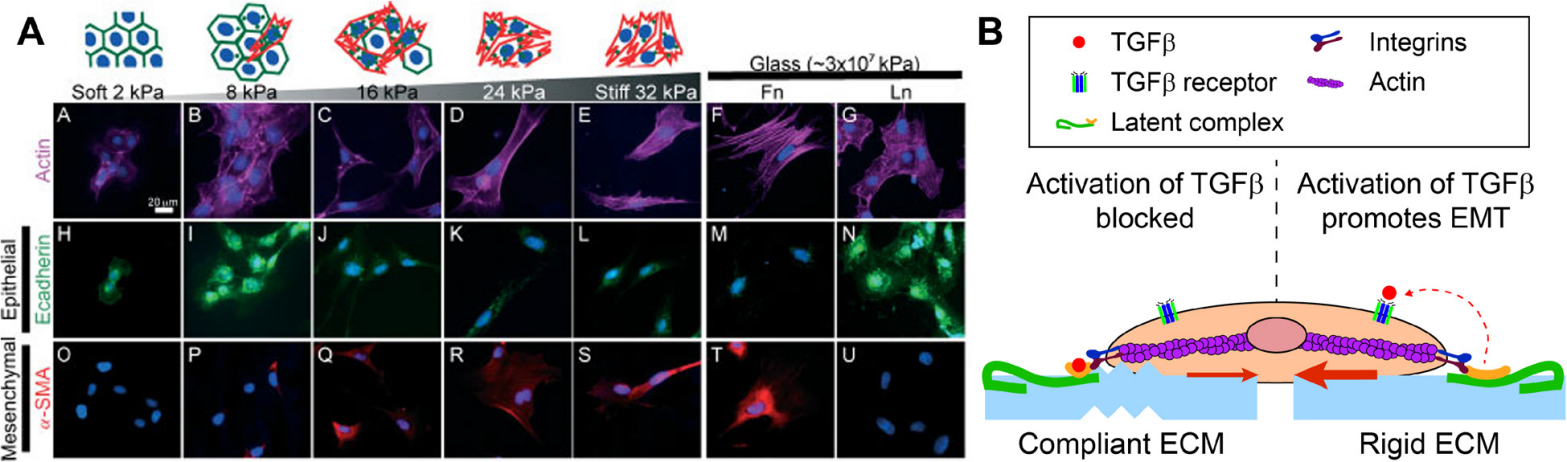
**Matrix rigidity promotes epithelial-myofibroblast transition. (A)** Immunofluorescence staining for actin, E-cadherin, and αSMA for primary alveolar type II cells cultured on fibronectin-coated polyacrylamide gels of varying rigidity or on fibronectin (Fn) or laminin (Ln) coated glass. The alveolar epithelial cells undergo EMT on rigid substrata. Panel **(A)** is from Brown et al, 2013 [147]. **(B)** Schematic depicting the activation of TGFβ from the latent complex. Adapted from Wells, 2013 [151]. Epithelial cells cultured on stiff matrices exhibit increased contractility thus enabling release of TGFβ from its latent complex thereby increasing the amount of active TGFβ accessible to bind to cell surface receptors.

Recent studies have identified matrix rigidity as a crucial regulator of TGFβ-induced EMT through several pathways [99,118,147,156]. Mammary and kidney epithelial cells exhibit a switch between TGFβ-induced apoptosis and EMT when cultured on compliant or rigid substrata, respectively [99]. In these studies, soft substrata blocked and rigid substrata promoted EMT regardless of whether the cells were cultured on fibronectin, collagen I, or recombinant basement membrane. The switch between apoptosis and EMT is controlled by activation of the PI3K/Akt signaling pathway, with increasing matrix rigidity promoting increased phosphorylation of Akt. Furthermore, cells cultured on rigid matrices are able to generate contractile forces which promote TGFβ activation from its latent complex by α_v_ integrins while compliant matrices block this process [118,119]. Activation of TGFβ on rigid substrata is promoted by Rho/ROCK signaling in lung epithelial cells and this induces EMT (Figure 3) [118,147,156]. On fibronectin-coated substrata, this response can be abrogated by culturing cells on rigid substrata coated with a fibronectin mutant which contains a stabilized RGD and PHSRN synergy site that supports α_3_ and α_5_ integrin engagement [156]. These results highlight the complex interplay between epithelial cells and both the chemical and physical properties of their microenvironment during induction of EMT. Moreover, these studies suggest that activation of EMT may create a positive feedback loop that enhances myofibroblast activation and ECM synthesis thereby further increasing the rigidity of the matrix and disease progression.

### Tissue geometry patterns EMT

During tissue development and wound healing, cellular behaviors are spatially patterned thus conferring cells at specific locations unique attributes and functions. Indeed, patterning within developing embryos is ensured in part by the temporal and spatial regulation of EMT [52]. Moreover, myofibroblasts have been observed at the edges of epithelial wounds [101,124] and pathological EMT and myofibroblasts are found along the invasion front of metastatic tumors [95,157,158].

Spatial variations in the mechanical properties of tissues are controlled by tissue composition and architecture as well as by the interaction of individual cells with the surrounding matrix and neighboring cells. Within epithelial tissues, neighboring cells exhibit cell-cell adhesions that are mediated by tight junctions and adherens junctions. These cell-cell junctions are functionally and dynamically connected to the actin cytoskeleton thus enabling transmission of forces between neighboring cells. In culture, intercellular transmission of mechanical stress through cell-cell adhesions can establish mechanical gradients with regions of maximal stress defined by the geometry of the tissue [102,159]. Spatial patterns in EMT can arise in two-dimensional epithelial sheets with downregulation of cytokeratins and upregulation of mesenchymal markers vimentin and αSMA occurring in regions of the tissue that experience the highest mechanical stresses [102]. The observed spatial patterning of TGFβ-induced EMT correlates with the subcellular localization of MRTFA, with EMT occurring in regions of the tissues with the highest frequencies of MRTFA nuclear localization.

### Cyclic stretch promotes EMT

Some cells within the body experience cyclic stretch during normal function, such as alveolar epithelia during respiration. Under conditions associated with fibrosis, epithelial cells may experience pathologically high levels of stretch arising from tissue distortion associated with injury or scar tissue formation. The effects of pathological levels of stretch on the induction of EMT have recently been highlighted in several studies. In a model system examining the pathological effects of renal tubular distension, kidney epithelial cells exposed to cyclic mechanical stretch exhibited increased EMT [160]. This effect was mediated by upregulation of TGFβ by more than two-fold in stretched cells in comparison to non-stretched cells. Cyclic mechanical stretch also promotes EMT in type II alveolar epithelial cells, not through upregulation of TGFβ, but rather by inducing actin polymerization and upregulation of low molecular weight hyaluronan which facilitates signaling through Wnt/β-catenin and MyD88 pathways [161]. Together, these studies demonstrate yet another important way in which mechanical cues can promote EMT and the fibrotic response of tissues to injury.

### Mechanosensitive signaling cascades in EMT

#### Myocardin related transcription factors

Acquisition of mesenchymal features during TGFβ-induced EMT is regulated in part by the SRF/MRTFA signaling pathway and we have highlighted several studies demonstrating the interplay between this pathway and mechanics in EMT. Thus far, a majority of studies examining this pathway in the context of EMT have focused on how MRTFA regulates the expression of cytoskeletal-associated genes such as αSMA. MRTFA also regulates the expression of EMT-associated transcription factors including Snail, Slug, and Twist [105] and therefore may have an impact on the expression of the epithelial gene E-cadherin. Further studies are necessary to define the role of MRTFA in the regulation of epithelial markers during EMT and to determine the impact of mechanical cues on the loss of epithelial features during EMT.

#### Hippo pathway

The Hippo pathway is critical for cell growth and cell fate decisions and dysregulation of signaling through this pathway or of its downstream effectors is implicated in fibrosis and cancer [162-165]. Downstream effectors in this pathway, Yes-associated protein (YAP) and transcriptional co-activator with PDZ-binding motif (TAZ), interact with the canonical TGFβ signaling cascade by regulating the subcellular localization of phosphorylated Smads [166,167]. Activation of these factors is controlled in part by cell-cell contact [167] and recent studies have demonstrated that YAP and TAZ mediate how cells respond to cell geometry and ECM elasticity to control cell growth and stem cell differentiation [168-171]. Indeed, cell shape and matrix rigidity modulate the subcellular localization of YAP and TAZ and cytoskeletal destabilization and inhibition of cell contractility inactivate YAP and TAZ [168]. TAZ is a critical regulator of local EMT at wound sites [172] and overexpression of TAZ can induce EMT [173]. Downregulation of TAZ blocks αSMA expression along wound edges and it has been suggested that TAZ may control αSMA expression either through association with MRTFA or through interaction with the αSMA promoter as a co-activator to the TEA domain (TEAD) transcription factors [172]. Given that YAP and TAZ are mechanosensitive and cytoskeletal architecture is linked to Hippo pathway signaling [174], it is plausible that mechanical signals control YAP and TAZ activity to regulate aspects of EMT. Future studies addressing the interplay of mechanical cues and YAP and TAZ signal transduction during TGFβ-induced EMT will be informative and may shed light on mechanisms mediating fibrosis and cancer.

### Targeting TGFβ-induced EMT

The multipotent nature of TGFβ signaling in normal and diseased tissues presents challenges for the development of therapeutics targeting this pathway. Nevertheless, much effort has been directed toward the development of antagonists of TGFβ [175-177]. Small molecule inhibitors are in various stages of development [177] and clinical trials are testing the efficacy of TGFβ monoclonal antibodies for treatment of diabetic nephropathy and idiopathic pulmonary fibrosis [58]. Furthermore, neutralizing antibodies against TGFβ have been found to reduce metastatic cancer progression in mice [178-182]. In addition, a promising approach which has demonstrated efficacy as an anti-fibrotic in lung, kidney, and liver disease models is targeting the integrin- and contractility-induced activation of TGFβ from it latent complex through the use of a monoclonal antibody to α_v_β_6_ integrin [183-186]. This method may also be an effective therapeutic approach for blocking tumor progression, as anti-α_v_β_6_ integrin monoclonal antibody prevents xenograft tumor growth *in vivo*[187]. Inhibiting TGFβ activation may present lower risk to the disruption of beneficial effects of TGFβ than targeting TGFβ itself since α_v_β_6_ is expressed primarily within epithelial cells and is highly upregulated in diseased tissues [184].

Targeting intracellular signaling cascades downstream of TGFβ rather than TGFβ itself may also be a viable approach for blocking fibrogenesis and cancer progression. Indeed, a recent study demonstrated that troglitazone, a peroxisome proliferator activated receptor (PPAR)-γ agonist that suppresses TGFβ-mediated fibrogenesis [188-190], attenuates TGFβ-induced phosphorylation of Akt and upregulation of Snail [97]. This is one of the major pathways activated within epithelial cells by the combination of TGFβ and matrix rigidity [99]. In addition, small molecule inhibitors including CCG-1423 and its analogs block SRF/MRTFA signaling [191,192]. Namely, CCG-1423 blocks the interaction of MRTFA with importin alpha/beta 1 thus preventing the nuclear import of MRTFA [193]. Furthermore, CCG-1423 has been shown to successfully inhibit TGFβ-induced expression of αSMA [102,104,194]. Interestingly, a recent study reported that the small molecule isoxazole can induce a myofibroblast phenotype by regulating the stability and activity of MRTFA [195]. Isoxazole enhanced cutaneous wound closure in mice suggesting that therapeutics aimed at promoting MRTFA signaling and the myofibroblast phenotype may also be promising methods for improving wound healing.

Given the link between TGFβ-induced EMT, fibrosis, and cancer, therapeutics directly targeting EMT may prove to be fruitful approaches for treating these diseases. Bone morphogenetic protein (BMP)-7 exhibits anti-fibrotic effects in animal models of renal fibrosis and reverses EMT in renal tubular cells *in vitro*[100,196]. Furthermore, a recent study found that a variety of anti-proliferative agents also inhibit EMT suggesting that the most effective compounds for cancer treatment may be those that target multiple aspects of cancer progression [197].

## Conclusions

The ability of epithelial cells to transition to a mesenchymal phenotype is regulated by cytokines, ECM components, cell-cell contacts, and mechanical cues and a combination of these factors is likely required for EMT induction. The studies highlighted within this review have identified an important role for mechanics in TGFβ-induced EMT and suggest that mechanical signaling pathways, including those involved in mechanotransduction, cell contractility, and regulation of matrix rigidity, could serve as potential targets for new therapies directed toward fibrosis and cancer. To achieve this though, a better understanding of the mechanistic underpinnings of how cell and tissue level physical properties contribute to EMT in pathological settings is needed.

## Abbreviations

AFM: Atomic force microscopy; BMP: Bone morphogenetic protein; ECM: Extracellular matrix; EGF: Epidermal growth factor; EMT: Epithelial-mesenchymal transition; FAK: Focal adhesion kinase; FGF: Fibroblast growth factor; Fn: Fibronectin; FSP-1: Fibroblast-specific protein-1; HAS2: Hyaluronan synthase 2; HGF: Hepatocyte growth factor; KLF8: Krüppel-like factor 8; Ln: Laminin; MAPK: Mitogen-activated protein kinase; MMP: Matrix metalloproteinase; MRTF: Myocardin related transcription factor; NMuMG: Normal murine mammary gland; PI3K: Phosphoinositide-3-kinase; PPAR: Peroxisome proliferator activated receptor; ROCK: Rho associated kinase; SRF: Serum response factor; TAZ: Transcriptional co-activator with PDZ-binding motif; TEAD: TEA domain; TGF: Transforming growth factor; YAP: Yes-associated protein; αSMA: Alpha smooth muscle actin.

## Competing interests

The authors declare that they have no competing interests.

## Authors’ contributions

JWO and EWG reviewed the literature, wrote, and revised the manuscript. Both authors read and approved the final manuscript.
